# Cellular information dynamics through transmembrane flow of ions

**DOI:** 10.1038/s41598-017-15182-2

**Published:** 2017-11-08

**Authors:** Robert A. Gatenby, B. Roy Frieden

**Affiliations:** 1 Departments of Radiology and Integrated Mathematical Oncology, Moffitt Cancer Center, Tampa, Florida 33612 USA; 2 College of Optical Sciences, University of Arizona, Tucson, Arizona 85721 USA

## Abstract

We propose cells generate large transmembrane ion gradients to form information circuits that detect, process, and respond to environmental perturbations or signals. In this model, the specialized gates of transmembrane ion channels function as information detectors that communicate to the cell through rapid and (usually) local pulses of ions. Information in the ion “puffs” is received and processed by the cell through resulting changes in charge density and/or mobile cation (and/or anion) concentrations alter the localization and function of peripheral membrane proteins. The subsequent changes in protein binding to the membrane or activation of K^+^, Ca^2+^ or Mg^2+^-dependent enzymes then constitute a cellular response to the perturbation. To test this hypothesis we analyzed ion-based signal transmission as a communication channel operating with coded inputs and decoded outputs. By minimizing the Kullback-Leibler cross entropy ${{\boldsymbol{H}}}_{{\boldsymbol{K}}{\boldsymbol{L}}}({\boldsymbol{p}}||{\boldsymbol{q}})$ between concentrations of the ion species inside ${{\boldsymbol{p}}}_{{\boldsymbol{i}}}({\boldsymbol{t}}){\boldsymbol{,}}{\boldsymbol{i}}={\boldsymbol{1}},\ldots ,{\boldsymbol{N}}$ and outside $\,{{\boldsymbol{q}}}_{{\boldsymbol{i}}}({\boldsymbol{t}})$ the cell membrane, we find signal transmission through transmembrane ion flow forms an optimal Shannon information channel that minimizes information loss and maximizes transmission speed. We demonstrate the ion dynamics in neuronal action potentials described by Hodgkin and Huxley (including the equations themselves) represent a special case of these general information principles.

## Introduction

Investigation of information dynamics in living systems typically focus on the role of the genome in transgenerational flow of information and, via epigenetics, variations in gene transcription in response to internal or external signals. Here we propose an additional mechanism of information dynamics that is based on ion fluxes at the cell membrane. As outlined below, we posit these dynamics are localized around the cell membrane and are independent of additional input from the genome, thus allowing both rapid and local responses to environmental perturbations or signals.

**Figure 1:**
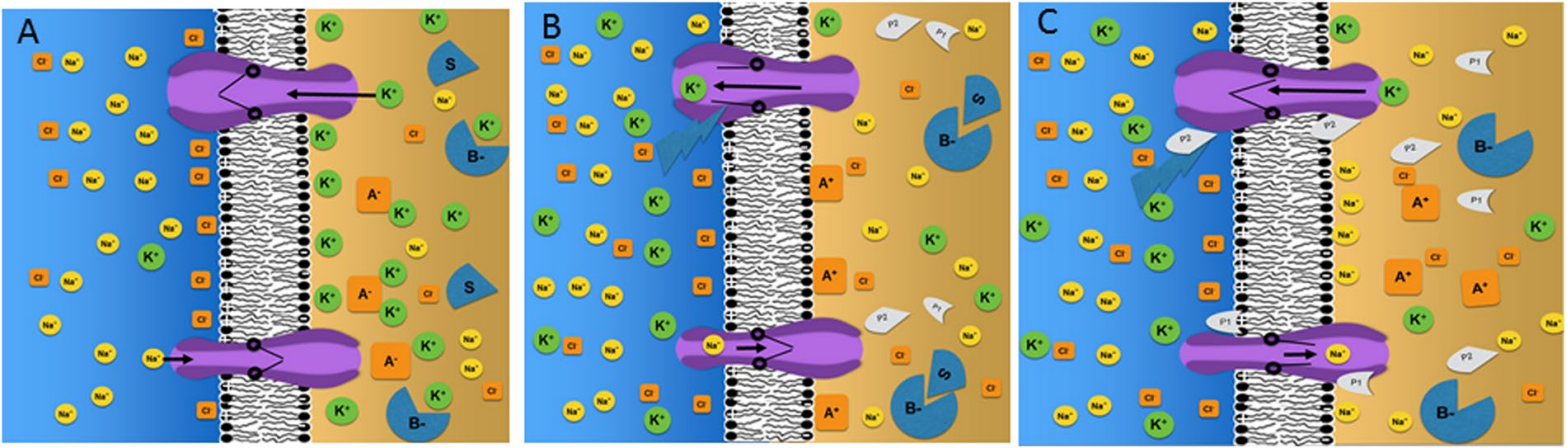
Summary of ion-based information dynamics around the cell membrane (**A**). Baseline state. The initial conditions of the cell membrane produced through energy-dependent ion pumps. Concentrations of Na^+^ and Cl^−^ are much higher in the extracellular space (*left*) than in the intracellular space (*right*) and vice versa for K^+^. Eukaryotic cells can use up to 40% of their energy budget to produce ion gradients. Specific transmembrane ion channels are present with their gates closed, thus allowing limited ion flow along concentration gradients. Peripheral membrane proteins included structural elements that support the membrane (proteins A) and enzymes (proteins B). Negative charges on enzymes (proteins B) are shielded by K^+^ ions causing the enzyme to be inactive. At the inner leaflet of the cell membrane primarily K^+^ ions shield proteins from the negative chargers of the membrane. As a result the positively charged protein A cannot bind. Note that K^+^ ions are about twice the  volume of Na^+^ ions. In our model, this subtle difference is posited to be sufficient to alter the shape of the active site and, therefore the activity of some enzymes (as shown in enzyme B). (**B**) Ion flux due to perturbation. An external cue causes the gate in the K^+^ channel to open such that K^+^ ions flow along concentration gradients out of the cell. This results in decreased shielding of the negative charges in the inner membrane leaflet so that positive charges on protein A can form electrostatic bonds. The binding of the structural protein to the lipid membrane stabilizes it as a response to the perturbation. Further, loss of K^+^ ions causes the enzyme (protein B) to change in both position and shape so that it binds to its substrate to catalyze the reaction S → P_1_ + P_2._ Note that biological enzymes can increase the reaction rate by as much as *15 orders of magnitude* allowing the response to be both local and rapid. (**C**) Ion-Flux Induces response to perturbation. Activation of the enzyme B results in rapid increase in the local concentrations of the reaction products P1 and P2. In this model, P2 blocks the actions of the perturbation closing the gate on the K channel. The other product, P1, opens the Na channel allowing rapid flow of cations, which restore the shielding on the inner leaf of the cell membrane, displacing the A proteins from the inner leaflet of the cell membrane back into the cytoplasm.

In brief, our hypothesis begins with the observation that virtually all cells generate transmembrane ion gradients produced by ATP-dependent membrane pumps. These gradients form high information^1,2^ states (*e.g*., large Shannon entropy) and possess high potential energy (similar to a coiled spring) initial conditions (Fig. 1A). External perturbations are detected by specific gates in the transmembrane ion channels. Information triggered by the external cue is then communicated to the cytoplasm through a local flow of ions that is controlled by opening the gates in these channels (Fig. 1B). The information from the “pulse” of ions (similar to Ca^2+^ puffs^3^) around one or more membrane channels is then translated into signaling circuits by changing local charge density and/or ion concentration. We note that the ion could flow into the cells creating the equivalent of the Ca^2+^ puffs or out of the cell generating a “negative puff” caused by absent ions (Fig. 1B).

The change in ions shielding negative charges on the inner leaf of the cell membrane can alter the binding of local peripheral membrane proteins^4^ thus altering the membrane structure and function (Fig. 1C). Similarly, opening of the potassium channel receptor by ligand binding to the Epidermal Growth Factor Receptor^5,6^ results in loss of K^+^ shielding of negative charges on the inner leaf of the cell membrane allowing “recruitment”^7,8^ of positively charged RAF proteins to the GTP-bound RAS proteins (which are also negatively charged).

An additional cellular response is elicited because the functions of many enzymes^9^ is dependent on local cation concentrations (e.g. Na^+^, Mg^2+^, Ca^2+^,or K^+^). The subsequent change in localization, activation or repression of local enzymes can produce an additional response to the local perturbation (Fig. 1C). When a successful response to the perturbation is complete, the ion gates close and the initial conditions (i.e. large transmembrane ion gradients) are restored to respond to any subsequent environmental changes.

These ion dynamics are similar to those observed in propagating action potentials of neurons^10^. However, we note that the steep transmembrane ion gradients that permit rapid flow of ions through the transmembrane channels of neurons are widely present in eukaryotes. Hence, we hypothesize that the transmembrane ion flows observed and modeled by Hodgkin and Huxley represent a special case of a broader phenomenon in which cells use transmembrane ion gradients to access, process and respond to information cues in the environment.

Here we assessed the potential of pulses of ions through cell membrane channels as a mode of information transmission, and define a general mathematical expression of these dynamics. We demonstrate that communication through ion fluxes represents an optimal mechanism for information transmission that both minimizes information loss and maximizes the speed of information transmission. Furthermore, our analyses support the notion that the ion dynamics observed in the Hodgkin-Huxley experiments and, indeed, the Hodgkin-Huxley equations themselves, represent a special case of these broader principles that are valid for a wide range of eukaryotic cells.

## Methods

### Changing ion concentrations around the cell membrane

Consider a problem of determining, over a time interval ${t}=({0},{\rm{\infty }})$, the generally varying change in the intracellular ion concentrations ${{p}}_{{i}}({t}),{i}={1},\ldots ,{N}$ of *N* types of ions that result from flow of ions through channels in the cell membrane. Examples might be the ions Na^+^, K^+^, Cl^−^, Ca^+2^, etc. Thus, ${{p}}_{{i}}({t})$ are the *probability density functions* over the *time* interval ${t}=({0},{\rm{\infty }})$ and so can also represent a rate or flow. We also use this terminology. Thus each type *i* of ion obeys normalization

1 $${\int }_{0}^{\infty }{p}_{i}(t)dt=1,$$

Also, definite boundary values $\,{{p}}_{{i}}({0}),{{p}}_{{i}}({\rm{\infty }})$ are taken to hold. What are the unknown fluctuations of ion probability density functions ${{p}}_{{i}}({t})$?

### Transmembrane ion flows as information

#### Constant extracellular charge density

Here we assume that, over the time domains in which a model functions, the probability densities of the ions outside of the membrane are fixed. That is, ATP-dependent membrane ions pumps will use energy to move ions into or out of the cell. This will have the effect of changing the intracellular ion concentrations but not those of extracellular ions ${{q}}_{{i}}({t})$, which are approximately constant

2 $${q}_{i}(t)\equiv {q}_{i}=constant,i=1,\ldots ,N,$$

in comparison to the generally varying probability densities ${{p}}_{{i}}({t})\,\,$of ions within the cell. Note that this constancy, or uniformity, is mathematically equivalent to the assumption of an ideal enzyme of uniform *density*, as is often used to characterize the Arrhenius law^11^.

Constancy (2) arises as follows:

- In the case of a single-cell organism the extracellular space is effectively the ocean, wherein ion concentrations do not change in biologically relevant time. Or
- For a multicellular organism, the extracellular space is in equilibrium with the interstitial fluid of the entire organisms (including, for example, the blood). Therefore the ion concentrations in the stable physiological state of a healthy individual should likewise tend to be constant. While there may be a small local changes in concentration exterior to the cell due to ion flow across the membrane, this will be quickly dissipated such that the extracellular ion space remains constant, serving as a source or sink for intracellular ions.

With the assumption that ${{q}}_{{i}}({t})\,\equiv\,{{q}}_{{i}}\,=\,{c}{o}{n}{s}{t}{a}{n}{t},\,{i}\,=\,1,\ldots ,{N},$ we can therefore assume that changes in intracellular ion concentrations ${{p}}_{{i}}({t}),{i}\,=\,{1},\ldots ,{N}$ are solely dependent on functions within the cell membrane. That is, membrane ion pumps generate transmembrane gradients by varying the intracellular ion concentrations relative to fixed extracellular concentrations. Further, when the channel gates open, the rapid flow of ions (measured to be about 10^5^ ions/second) of ions allows ${{p}}_{{i}}({t})\to {{q}}_{{i}}$. *We propose this ion-based communication channel represents a method for information transfer that both minimizes loss and maximizes speed*..

#### Criterion of minimum information loss

We first seek a general solution ${{p}}_{{i}}({t}),{i}={1},\ldots ,{N}$ for intracellular ion concentration changes that is consistent with the constant concentrations ${{q}}_{{i}}$ outside the cell. We first useful to consider how informative flow rates ${{p}}_{{i}}({t})$ occur.

We start with the assumption that ATP-dependent membrane pumps transfer ions across the cell membrane. Because of these ion-specific pumps, the intracellular concentrations of Na^+^ and Cl^−^ are much lower than those in the extracellular space, and vice versa for the concentrations of K^+^ (Fig. 1). The non-equilibrium condition is manifested by an often large transmembrane potential *V*. Notably, the asymmetric distribution of ions produced by these pumps (compared to random distribution in the absence of those pumps) encodes on the order of 10^14^ bits of Shannon^12^ information^1,2,13,14^.

Suppose the flow of some number of ions from outside to inside the cell membrane can signal the occurrence of an environmental event. For this to occur, the event must trigger the gates in one or more of the ion-specific membrane channels. These gates permit ion flow along the pre-existing concentration gradients between the inside and outside of the cell. Several hundred types of channel gates are encoded in the human genome, permitting responses to a potentially large range of external perturbations. The role of ion flow in cell functions is most well-studied in neurons. To propagate nerve impulses, the channel gates along the axis of the neuron respond to voltage fluctuations cause by the local arrival of the action potential. When the gates open, the transmembrane gradient permits rapid ion flow along steep concentration gradients producing the action potential described by the Hodgkin Huxley equations.

We assume that evolution selects for optimal means for addressing risk and capitalizing on opportunities in the environment. We propose that this optimization principle selects for information dynamics at the membrane, which conveys maximum Shannon information regarding the *occurrence times* of these events. Of course the system is passive so that, in general, there can only be a loss of such information relative to the constant ion density probability functions ${{q}}_{{i}},{i}={1},\ldots ,{N}$ *outside*. Accordingly, *the maximal Shannon information is* conveyed when the resulting intracellular ion probability density $\,{{p}}_{{i}}({t})$ is minimally different the ${c}{o}{n}{s}{t}{a}{n}{t}\equiv {{q}}_{{i}}({t})\equiv {{q}}_{{i}}$ of Eq. (2). As noted below, this also defines how fast the information from an environmental source can be decoded by the cell surface.

### Acceleration of transmembrane ion flows through gated channels

A reasonable assumption is that an environmental change is sensed at the cell membrane. This could represent a change in a physical interaction with an adjacent cell or other surface or a change in the local concentration of some substance (*e.g*., substrate molecules in the case of a free swimming eukaryote or, in the case of neurons, a neurotransmitter). How is this information communicated into the cell? We propose that eukaryotic cells have efficient mechanisms for sensing and reacting to environmental change through *gated ion channels* on the cell membrane. ATP-dependent membrane ion pumps typically generate large transmembrane gradients but the flow of ions along such gradients is ordinarily very slow (“leakage current”) due to high resistance in nonpolar regions of the cell membrane. Opening of channel gates by an external perturbation has the effect of greatly expanding the transmembrane flow of the specific ion that passes through that channel. Indeed, it has been noted that the actions of the channel in the membrane are analogous to a catalyst^15^ for diffusion, in the sense that the acceleration of the ion flow are qualitatively and quantitatively similar to enzyme-induced acceleration of a chemical reaction rate.

What can we learn by considering such an analogy? As will be shown, it leads to derivation of the Hodgkin-Huxley equations that govern the propagation of ions across the neural cell membrane. Moreover, *the derivation is sufficiently general to apply to ion flow through gates in a wide class of cell membranes*.

In general the passage of a given type of ion, say K^+^, is restricted to a specific channel just as each of the key ions (*e.g*., Na^+^, K^+^, Cl^−^, Ca^2+^, Mg^2+^) can pass through one and only one type of channel^16^. Each such ion then represents a distinct component of the transmembrane information flow. The channels contain gates that are ordinarily closed, thus preventing ion flow. However, this still allows a “leakage” current, which is the usually small flow of ions through the membrane. Once a transmembrane ion gradient is established through ATP-dependent membrane ion pumps, maintenance of that gradient requires only that the pumps replace the small loss of ions due to leakage current. A detailed description of the gate channels can be found in^16^.

Finally, the passage of a given type of ion, say K^+^, requires that more than one gate in the channel opens. For example, the K^+^ channel allows ion flow only when all 3 channel gates are open. These appear to form a ring shaped gap through which the required K^+^ ion passes^17,18^.

### Principle of minimum Kullback-Leibler divergence

The cell membrane provides a ‘window’ to the world outside for each cell. Some signals that arrive at the membrane, such as growth factors, require transmission through well recognized cytoplasmic signaling pathways to the nucleus for further processing and action. However, others, particularly life-threatening perturbations, may require an immediate response that is mediated entirely by the molecular machinery in and around the cell membrane.

The rapid transmembrane flow of ions within one or more channels allows cells to receive, locally process and respond to information in the environment. Thus, ions of type *i* = 1*,.., N* may pass through the cell membrane, each potentially signaling vital information to the cell. Hence the changing concentration, and correct identification, of the various ion types is vital to cell survival and proliferation. We shall assume that *natural selection*, acting through geological time, has optimized the speed and accuracy of identifying *i* on average. This implies that the intracellular probability functions ${{p}}_{{i}}({t})$ are formed, coded, and decoded at the membrane according to a well-defined optimization principle. We use minimum average code length as the criterion^19^, as follows (in brief, see Eqs (3), (4)).

### Information flow optimization by maximally efficient code

Claude Shannon^12,20^ posed the following question of optimization: If we are given a probability ${{q}}_{{i}}({t})$ on event times *t* for events *i*, what code lengths ${{l}}_{{i}}({t})$, *i* = *1*, .., *N* achieve their minimum expected values $< {{l}}_{{i}}({t}) >$ over all times *t*? This defines a maximally efficient code. Shannon and Fano showed that if symbols (say letters of an alphabet) ${{a}}_{{i}}$, *i* = *1*, .., *N* occur with respective frequencies ${{q}}_{{i}}$ these are coded with maximum efficiency (minimum average code length) if each symbol *i* is coded with a length ${{l}}_{{i}}=\,{\rm{l}}{\rm{o}}{\rm{g}}(\frac{{1}}{{{q}}_{{i}}})$. Thus, the most frequently occurring symbols ${{a}}_{{i}}$ would be coded with minimum lengths *l*. Or, minimum code lengths are required, and this is independent of time. This is intuitive for maximally fast response. Thus, the information dynamics at the cell membrane in which the external ion concentrations (expressed as probabilities ${{q}}_{{i}}({t})$) are *actually* constants ${{q}}_{{i}}$, manifest the conditions for optimum coding.

However, the same constancy does not hold in practice for data. Their occurrence rates ${{p}}_{{i}}({t})$ randomly differ rates from the ideal rates ${{q}}_{{i}}$. (Think of the letter rates on a single page of print). Hence, if these are coded according to Shannon and Fano’s scheme, the result is a wasteful *excess* of code lengths by amount

3 $${l}_{q}(t)\,-\,{l}_{i}(t)=\,\mathrm{log}(\frac{1}{{q}_{i}})\,-\,\mathrm{log}(\frac{1}{{p}_{i}(t)})=\,\mathrm{log}(\frac{{p}_{i}(t)}{{q}_{i}})$$

at each time. The expected value of the *excess* code length *over all time* for each ion type *i* is then $< {\rm{l}}{\rm{o}}{\rm{g}}(\frac{{{p}}_{{i}}({t})}{{{q}}_{{i}}}) >$. This average excess code length translates into excessive total average communication time required for *responding to the environmental event* being reported on by the ion. In turn, this implies excess reaction time for coping with that event. Excess reaction time to an external threat, for example, can be deadly for the cell. Hence we assume that natural selection minimizes reaction time.

#### The role of multiple channels

The above models focus on a single specific channel and channel state. This is clearly an oversimplification as it is likely that ion flows into mammalian cells are the result of an ensemble of single channels that superimpose at various times with various weights to form the net channel state^21^. This may vary with time both stochastically and with subtle changes in the environmental condition. These ensemble dynamics leading to a net channel can be accommodated using KL principle because it is a linear differential equation. Thus, the net channel state *P*_*n*_(*t*) is, by hypothesis, the statistically average state. Therefore any linearly weighted superposition of single channel outputs will also satisfy our KL principle so that the net state *P*_*n*_(*t*) obeys KL principle. However, the detailed shape of the curve *P*_*n*_(*t*) will depend upon what the times ${\tau }_{n}$, *n* = *1*, …, *4* are in the various channel states. Nevertheless, even allowing for a quite complex ensemble of independent channels, the total ion flux gives an output activation wave obeying the KL principle.

#### Role of natural selection

The average such excess is assumed to be minimized, as an expression of natural selection (see above). The average is evaluated by simply weighting the r.h.s. of (3) with ${p}_{i}(t)$ and integrating over all *t*, as

4 $${\int }_{0}^{\infty }{p}_{i}(t)\mathrm{ln}(({p}_{i}(t)/{q}_{i})dt\equiv {H}_{KL}({p}_{i}||{q}_{i})=minimum,\,i=1,\ldots ,N.$$

In words, Eq. (4) states the average amount of *wasted code length* over all ion occurrences *i* equals the relative entropy ${H}_{KL}(p||q)$ or the ‘Kullback–Leibler divergence^22^. This is effectively a distance between probabilities ${{p}}_{{i}}({t})\,\,$and ${q}_{i}$, ${i}={1},\ldots ,{N}$. For example, at any *i* and *t* for which probability ${{p}}_{{i}}({t})={{q}}_{{i}}$, there is contribution ${\rm{l}}{\rm{n}}({1})={0}$ to ${H}_{KL}({p}_{i}||{q}_{i})$ in Eq. (4). There is thus zero distance between such pairs. This implies that if probabilities ${p}_{i}(t)$ are formed, coded, and decoded at the membrane according to principle (4) the external environmental events will be identified by the cell membrane with minimum error and maximum speed. We propose that these optimization principles, because the allow maximally fast and accurate responses to external perturbations,  are fundamental Darwinian selection forces in the evolution of all cells.

Quite aside from the above requirement by natural evolution, the minimization requirement in Eq. (4) is also suggested by the dual facts that (i) the Kullback–Leibler divergence Eq. (4) has the form of the *Shannon information* ^12^, and (ii) the transmission of the ion through the membrane is an irreversible thermodynamic process which, therefore, obeys coarse graining^23–26^. Such processes can only lose information, and so we again arrive at principle (4) which states that optimal function requires this loss of information to be a minimum^27^.

We note that the Kullback–Leibler principle Eq. (4) is being used as a principle of thermodynamics. Such use allows microscale effects, for example occurrence rates ${{p}}_{{i}}({t})$, to be found without detailed knowledge of that microscale, such as the mathematical dependences of the fields of potential (*e.g*., inverse square) that are provided by ions. Thermodynamics is thereby seen to provide a basis for the phenomenon of natural selection.

#### Convex nature of ${{H}}_{{K}{L}}$

The minimum in principle (4) is always an extreme value since ${{H}}_{{K}{L}}({{p}}_{{A}}||{{q}}_{{A}})$ is a convex functional. This is fortunate, as finding its extreme value is then elementary (see examples below).

In summary to this point, the solution to principle (4) is found under the special condition Eq. (2) of a constant ${{q}}_{{i}}({t})\equiv {{q}}_{{i}}$. Also the solution ${{p}}_{{i}}({t})$ obeys:

(i) minimum departure from that of the constant ion rate ${{q}}_{{i}}$ (of Eq. (2)) exterior to the cell, in the sense of carrying *a level of Shannon information that departs minimally* from that of the constant ${{q}}_{{i}}$, and

(ii) optimally efficient information transfer of environmental messages (such that they are decoded with maximum speed within the membrane).

#### Tendency for solution to obey ${{p}}_{{i}}({t})\approx {{q}}_{{i}}({t})$

Note that in Eq. (4) that at all *t* for which ${{p}}_{{i}}({t})\,\approx \,{{q}}_{{i}}({t}),\,\,$since ${\rm{l}}{\rm{n}}({1})={0}$ there is a very small contribution to ${H}_{KL}$. Conversely, for all *t* for which ${{p}}_{{i}}({t})\,\gg \,{{q}}_{{i}}({t})$ there is large contribution to ${H}_{KL}$. Thus, mathematically, ${H}_{KL}({p}_{i}||{q}_{i})$ is a type of logarithmic measure of the ‘distance’ between corresponding ${{p}}_{{i}}$ and ${{q}}_{{i}}$ over all ${i}={1},\ldots ,{N}$. (Note: ${{H}}_{{K}{L}}$ isn’t a formal ‘distance’ measure as it isn’t symmetric in exchange of corresponding ${{p}}_{{i}}$ and ${{q}}_{{i}}$, but this is not important for our purposes, as will become apparent).

#### Use of prior knowledge

The minimization in principle (4) is always found for a given state of prior knowledge, usually in the form of equality constraints on the ${p}_{i}(t)$. These are implemented by the simple weighted addition of the constraints to principle (4), where each is multiplied by a corresponding constant Lagrange multiplier constant. These constants are found by requiring them to obey the known constraints (see below).

### Data Availability

No datasets were generated or analyzed during the current study.

## Results

### Application of the principle to membrane ion channels

The membrane is regarded to generally have some level of transmembrane potential *V* that can change with time *t*. Hence principle (4) is applied for some arbitrary values of *V*_*t*_. In addition, the minimum in Eq. (4) is attained in the presence of known constraints obeyed by the unknown laws ${{p}}_{{i}}({t}),{{q}}_{{i}}$. In general, these obey two, trivial normalization constraints, which are obeyed in all problems plus other, more substantive constraints such as mean passage time through the membrane. These effectively define the particular problem at hand.

We next compared results with known experimental observations, specifically of those of ions that pass through membrane ion channels in the membrane of a neuron – as described by the classical Hodgkin-Huxley equation. Hence, let the law ${{p}}_{{i}}({t})$ obey substantive constraints as follows. As with Hodgkin and Huxley^10^ let the ions pass through the cell membrane over fixed average times ${\tau }_{{i}}$,$\,{1},\ldots ,{N}$. These ${\tau }_{{i}}$, were empirically observed by Hodgkin and Huxley. It is then seen that the particular values of ${\tau }_{{i}}$ are substantive in strongly influencing the output rates ${{p}}_{{i}}({t})$ obeying principle (4).

Hodgkin and Huxley investigated, in particular, the case of *N* = *3* types of ions, specifically Na^+^, K^+^ and Cl^−^. They observed average path times ${\tau }_{{i}},\,{i}={1},\,{2},{3}$ for these types of ions. Hence, these are used as constraints on the time behavior determined by principle (4). As is usual, these constraints are entered via Lagrange constraint multipliers ${\lambda }_{{i}},\,{i}\,=\,{1},{2},{3}$. Constancy effect Eq. (1) and normalization Eq. (2) are also so used. The resulting constrained principle (4) is

5 $${\int }_{0}^{T}{p}_{i}(t)\mathrm{ln}(({p}_{i}(t)/{q}_{i})dt+{\lambda }_{1}[{\int }_{0}^{T\,}dt{p}_{i}(t)-1]+{\lambda }_{2}[{\int }_{0}^{T}dt{q}_{i}-1]+{\lambda }_{3}[{\int }_{0}^{T}dtt{p}_{i}(t)-{\tau }_{i}]=min$$

We will see that this principle gives rise to the known dynamical equations of Hodgkin and Huxley for occurrence rates ${{p}}_{{i}}({t})$ at the assumed potential value *V*.

#### Derivation

We note the absence of any derivative terms ${d}{p}{/}{d}{t}$ in the integrands of Eq. (5). Then its Euler-Lagrange solution simplifies to that obtained by simply setting partial derivative ${\rm{\partial }}{/}{\rm{\partial }}{{p}}_{{i}}$ of its Lagrangian (total integrand) to zero. This gives a requirement

6 $$1+ln{p}_{i}\,-\,ln{q}_{i}+{\lambda }_{1}+{\lambda }_{3}t=0$$

at the arbitrary time *t*. Solving for ${{p}}_{{i}}$,

7 $$\,{{p}}_{{i}}\,({t})={{q}}_{{i}}{e}{x}{p}(-{1}{-}\,{\lambda }_{{1}}\,{-}\,{\lambda }_{{3}}{t})$$

Boundary values ${{p}}_{{i}}({0}),{{p}}_{{i}}({T})$ were likewise observed by H and H. These should be obeyed by solution (7). Enforcing these in (7) gives the following solutions.

8 $${p}_{i}(t)={p}_{i}(T)\,-\,{q}_{i}exp(-1\,-\,{\lambda }_{1})\exp (-{\lambda }_{3}t),{q}_{i}=const.$$

Evaluating this at *t* = 0 gives

$${p}_{i}(0)={p}_{i}(T)-{q}_{i}exp(-1\,-\,{\lambda }_{1})$$

Solving,

$${q}_{i}exp(-1\,-\,{\lambda }_{1})={p}_{i}(T)\,-\,{p}_{i}(0)$$

Putting this in (8) then gives

9a $${p}_{i}(t)={p}_{i}(T)\,-\,({p}_{i}(T)\,-\,{p}_{i}(0))\exp (-{\lambda }_{3}t)$$

#### Evaluating Lagrange constants ${\lambda }_{{1}},{\lambda }_{{3}}$

In principle (5), the mean time constraint ${\int }_{{0}}^{{T}}{d}{t}{t}{{p}}_{{i}}({t})={\tau }_{{i}}$ and normalization constraint

$${\int }_{0}^{T}{p}_{i}(t)dt=1$$

giving the result

9b $${\int }_{0}^{T}t{p}_{i}(t)dt={\lambda }_{3}=1/{\tau }_{i}$$

Then result (9a) becomes

10 $${p}_{i}(t)={p}_{i}(T)\,-\,({p}_{i}(T)\,-\,{p}_{i}(0))\exp (-\frac{t}{{\tau }_{i}}),\,i=1,\ldots ,N.$$

These are equivalent to the Hodgkin and Huxley (H and H) equations numbered (8), (17) and (18) in^10^. Thus, the dynamics described by the “Hodgkin and Huxley” equations apply to a wider scope of cell membranes than merely neurons. As a check, we compare Eq. (10) with Eq. (8) of H and H. In their notation this is

11 $$n={n}_{\infty }-({n}_{\infty }-{n}_{0})\exp (-\frac{t}{{\tau }_{i}})$$

Thus our notation for the *i*th rate ${{p}}_{{i}}\,\,$in Eq. (10) becomes simply *n* for H and H, with their subscript denoting the time. Hodgkin and Huxley take their end time *T* to be very large, effectively $\,\infty$. Thus our notation ${{p}}_{{i}}({T})$ translates into ${{n}}_{{T}}\equiv {{n}}_{{\rm{\infty }}}$ in their Eq. (8) [Eq. (11) preceding]. Also as in Hodgkin and Huxley^14^, a ‘resting state’ is defined by *V* = 0. Let this occur at *t*ime *t* = 0. Then ${{p}}_{{i}}({0})$ in Eq. (10) becomes ${{n}}_{{0}}$ in their Eq. (8) [same as preceding Eq. (11)], although the argument 0 in ${{p}}_{{i}}({0})$ explicitly represents their case of zero potential.

#### Some consequences of flow solution

Note that if all time constants ${\tau }_{{i}}$ in Eq. (10) are very large this gives ${{p}}_{{i}}({t})\approx {{p}}_{{i}}({0}),$ no environmental information significantly enters the cell. However, such large ${\tau }_{{i}}$ have their use. As noted above, passage of any ion across the membrane requires that a specific number of gates open through which the ion then passes. For example, passage of a K^+^ ion through a membrane channel will not occur until all 3 gates open. The latter probability is of approximate size ${{p}}_{{{K}}_{+}}{({0})}^{{3}}$. Alternatively, the passage of an Na^+^ ion requires that 2 gates in the ion channel open, which then has probability$\,{{p}}_{{N}{a}+}{({0})}^{{2}}$. As the square of a probability tends to be greater than the cube of a comparable one, the Na^+^ ions are more probable to pass through the channel than are 3 K^+^ ions. Consequently, a cell tends to efflux Na^+^ before influxing K^+^. Further, there is a need for this time gap between the two since, otherwise, the passages of the two ions might so overlap in time such that they collide with one another. Thus, it is important for K^+^ ions to be delayed in their passage through the membrane. Collectively, the flow solutions both accelerate and slow selected ions to optimize decoding times and avoid collisions.

### Ion transition rate constants *α*_*m*_, *β*_*m*_

Probabilities ${{p}}_{{i}}({0}),{{p}}_{{i}}({\rm{\infty }}),\,{i}={1},\ldots ,{N}$ must be further defined. This involves the vital role played by the membrane potential *V*.

Ion transitions results from opening of channel gates that are governed by a perturbation in the transmembrane voltage *V*. However, as in the preceding, at ${t}={0}$ one assumes no perturbation is applied to the membrane so voltage ${V}={0.}$ Also, let ${\alpha }_{{m}}({0}),{\beta }_{{m}}({0})$ represent, respectively, the rate of ion transfer at this voltage from outside the cell membrane to inside and from inside to outside. This represents slow “leakage”^10^ current with a probability

12 $${p}_{i}(0)=\frac{{\alpha }_{i}(0)}{{\alpha }_{i}(0)+{\beta }_{i}(0)}.$$

Next, we have assumed that a fixed, finite voltage *V* is applied to the membrane due to arrival of a propagating wave down the axon of the nerve. Then rates ${\alpha }_{{i}}={\alpha }_{{i}}({V}),{\beta }_{{i}}={\beta }_{{i}}({V})$ instantly take on *fixed* values (1), appropriate to the value of *V over time t* that represents the course of the action potential. This allows us to suppress all *V* notation (as in ref.^10^). Thus in particular

13 $${p}_{i}(\infty )=\frac{{\alpha }_{i}}{{\alpha }_{i}+{\beta }_{i}}.$$

Endpoint values (12) and (13) supplement the interior H-H solution Eq. (10). Finally, by the above definition of ${\alpha }_{{i}},$ with V fixed and finite, it must be that the out-to-in ion rate obeys ${\alpha }_{{i}}={{p}}_{{i}}({\rm{\infty }}){/}{\tau }_{{i}}$ and the in-to-out rate obeys ${\beta }_{{i}}=\frac{{1}{-}{{p}}_{{i}}({\rm{\infty }})}{{\tau }_{{i}}}.$ Adding these gives

14 $${\tau }_{i}=\frac{1}{{\alpha }_{i}+{\beta }_{i}},\,i=1,\ldots ,N.$$

This shows how the rates ${\alpha }_{{i}},{\beta }_{{i}}$ determine the rapidity of exponential change of ion rate ${{p}}_{{i}}({t})$ in Eqs (10).

These rates obviously play a similar role in our solution Eq. (10), supplemented by Eq. (14), to that of the activation energy ${{E}}_{{a}}$ in the Arrhenius law^13^. They both govern the rapidity of an exponential falloff.

## Discussion

In summary, we propose the transmembrane ion gradients that permit neurons to form and propagate an action potential also play a critical role in the function and survival of non-neuronal cells. We propose this difference between extra- and intra-cellular ion concentrations produces initial conditions that allow reception, communication, processing and response to environmental information (Fig. 1). The information is detected by specialized gates within transmembrane ion channels that, by opening, allow transmission of the information into the cell in the form of an ion puff. By changing the structure of the proteins that form the gates, they can be made to be sensitive to a wide range of environmental factors. Furthermore, the information encoded in the flow of ions is received and processed in the cell through a local change of ion density and cation/anion concentrations around the channel. This may reduce (or increase) the mobile ion screening of negative charges in the inner leaf of the cell membrane and by increasing (or decreasing) the Debye length^28,29^, alter the attachment of peripheral membrane proteins (Fig. 1B). This can alter the structure of the membrane to respond to the perturbation. Furthermore, the function of a large number of enzymes is dependent on local is dependent of the local ion concentrations. For example, hexokinase^30,31^ is dependent on local Mg^2+^ concentrations and pyruvate kinase^9^ is dependent on K^+^ concentrations. In this way, the ion flux through K^+^ or Mg^2+^ ^32^ channels can promote or suppress reactions to rapidly respond to a perturbation.

Since the discovery of the structure and function of DNA, investigations of biological information dynamics have focused almost exclusively on gene replication and expression, and on translation. These include transgenerational transmission of information through chromosomes, epigenetic control and time dependent changes in transcription of genetic information in response to local environmental changes. However, in prior theoretical studies we have noted that the transmembrane ion gradients potentially may encode in excess of 10^14^ bits of Shannon information^1,13,14^. Further, the membrane ion protein pumps that generate these gradients can consume up to 40% of a cell’s energy budget^33^. Why does a cell invest such a large fraction of its resources to the formation of a transmembrane ion gradient? Indeed, at first glance, the large expenditure of energy to achieve the seemingly trivial task of replacing intracellular Na^+^ by K^+^ (or replacing Ca^2+^ with Mg^2+^), seems at best puzzling.

Here we propose a specific mechanism by which the transmembrane ion gradients, and their associated information dynamics, can detect, process and respond to environmental perturbations on a very rapid time scale without any direct input from the genes. That is, while the genome clearly contributes to these dynamics by encoding and translating the information in the associated proteins, the ion-based dynamics at the membrane can proceed locally and rapidly without additional input from the nucleus. As noted above, this model of information dynamics optimizes both speed and accuracy of information transmission. Thus, we propose that the large resource expenditure necessary for generation of the ion gradient is an indication of the critical role of these information dynamics in cell survival and function.

In general, our results support a more distributive model of biological information. Thus, information dynamics at the cell membrane are deeply linked with heritable information which contributes the molecular machinery that generates the gradients and permits detection, processing and response to environmental signals and perturbations. Similarly while we view the membrane information dynamics to occur independently on fast and local scales, we note that ion fluxes can generate molecular signals including lipid second messengers^34^ that communicate with the nucleus and other cellular organelles. Furthermore, ion flows may facilitate direct communication of cellular information to the nucleus. For example, ligand binding to EGFR is associated with gate opening in K^+^ channels^5,35^. This loss of K^+^ screening for the negative charges in the inner leaf of the membrane (Fig. 1A,B) may facilitate transmission of signal along the MAPK pathway by allowing coulomb interactions between positively charged Raf protein (pK of 9.3) or its associated scaffolding proteins to the GTP-activated Ras protein. Indeed, it is often stated that during MAPK signaling that Ras “recruits”^7^ Raf to the membrane. We note that the term “recruits” has no physical meaning but rather represent empirical observations that Raf moves very rapidly to the location to the membrane site containing an activated Ras. We propose the ion dynamics summarized in Fig. 1 could account for this rapid and specific movement.

## References

[1] Gatenby RA, Frieden BR (2005). The role of non-genomic information in maintaining thermodynamic stability in living systems. Math Biosci Eng.

[2] Gatenby RA, Frieden BR (2013). The Critical Roles of Information and Nonequilibrium Thermodynamics in Evolution of Living Systems. B Math Biol.

[3] Miyazaki K, Ross WN (2013). Ca2+ sparks and puffs are generated and interact in rat hippocampal CA1 pyramidal neuron dendrites. J Neurosci.

[4] Morozova D, Guigas G, Weiss M (2011). Dynamic structure formation of peripheral membrane proteins. PLoS Comput Biol.

[5] Bowlby MR, Fadool DA, Holmes TC, Levitan IB (1997). Modulation of the Kv1.3 potassium channel by receptor tyrosine kinases. J Gen Physiol.

[6] Zwick E (1997). Critical role of calcium- dependent epidermal growth factor receptor transactivation in PC12 cell membrane depolarization and bradykinin signaling. J Biol Chem.

[7] Marais R, Light Y, Paterson HF, Marshall CJ (1995). Ras recruits Raf-1 to the plasma membrane for activation by tyrosine phosphorylation. EMBO J.

[8] Stokoe D, Macdonald SG, Cadwallader K, Symons M, Hancock JF (1994). Activation of Raf as a result of recruitment to the plasma membrane. Science.

[9] Page MJ, Di Cera E (2006). Role of Na+ and K+ in enzyme function. Physiol Rev.

[10] Hodgkin AL, Huxley AF (1952). A quantitative description of membrane current and its application to conduction and excitation in nerve. J Physiol.

[11] Backhurst, J. R., Harker, J.H. & Richardson, J. F. *Coulson JMCe. Coulson & Richardson’s chemical engineering, J.M. Coulson and J.F. Richardson: solutions to the problems inChemical engineeri*ng, volume 2 (5th edition) and volume 3 (3rd edition). Butterworth-Heinemann (2002).

[12] Shannon CE (1997). The mathematical theory of communication. 1963. MD Comput.

[13] Gatenby R, Frieden BR (2016). Investigating Information Dynamics in Living Systems through the Structure and Function of Enzymes. PLoS One.

[14] Gatenby RA, Frieden BR (2007). Information theory in living systems, methods, applications, and challenges. B Math Biol.

[15] Eisenberg RS (1990). Channels as Enzymes: Oxymoron and Tautology Topical Review. Journal of Membrane Biology.

[16] Alberts, B. *Molecular biology of the cell*, Sixth edition. edn. Garland Science, Taylor and Francis Group (2015).

[17] Colatsky, T. J. *Potassium channels: basic function and therapeutic aspects: proceedings of the 29th Annual A.N. Richards Symposium held at Valley Forge, Pennsylvania, May 16–17, 1988*. Wiley-Liss (1990). 2309004

[18] Lippiat, J. D. *Potassium channels: methods and protocols*. Humana;Springer, distributor (2008).

[19] Barron A, Rissanen J, Yu B (1998). The Minimum Description Length Principle in Coding and Modeling. IEEE Transactions on Information Theory.

[20] Shannon CE (1998). Communication theory of secrecy systems. 1945. MD Comput.

[21] Rudy Y, Silva JR (2006). Computational biology in the study of cardiac ion channels and cell electrophysiology. Q Rev Biophys.

[22] Kullback SL, On RA (1951). information and sufficiency. Annals of Mathematical Statistics.

[23] Katchalsky A (1962). Kedemo. Thermodynamics of flow processes in biological systems. Biophys J.

[24] Katzir-Katchalsky, A. & Curran, P. F. *Nonequilibrium thermodynamics in biophysics*. Harvard University Press (1965).

[25] Kondepudi, D. & Prigogine, I. *Modern thermodynamics: from heat engines to dissipative structure*s, Second edition. edn. John Wiley & Sons Inc. (2015).

[26] Hsieh CP (2017). Nonequilibrium Thermodynamics of Ion Flux through Membrane Channels. Entropy.

[27] Csiszár I (2008). Axiomatic Characterizations of Information Measures. Entropy.

[28] Ding M, Lu BS, Xing X (2016). Charged plate in asymmetric electrolytes: One-loop renormalization of surface charge density and Debye length due to ionic correlations. Phys Rev E.

[29] Bukar N, Zhao SS, Charbonneau DM, Pelletier JN, Masson JF (2014). Influence of the Debye length on the interaction of a small molecule-modified Au nanoparticle with a surface-bound bioreceptor. Chem Commun (Camb).

[30] Ouwerkerk R, van Echteld CJ, Staal GE, Rijksen G (1989). Intracellular free magnesium and phosphorylated metabolites in hexokinase- and pyruvate kinase-deficient red cells measured using 31P-NMR spectroscopy. Biochim Biophys Acta.

[31] Norton GE, Feldman I (1980). Effects of free magnesium and alkali ions on the conformation and glucose-binding strength of yeast hexokinase isozymes. Biochim Biophys Acta.

[32] Payandeh J, Pfoh R, Pai EF (2013). The structure and regulation of magnesium selective ion channels. Biochim Biophys Acta.

[33] Guppy M, Kong SE, Niu X, Busfield S, Klinken SP (1997). Method for measuring a comprehensive energy budget in a proliferating cell system over multiple cell cycles. J Cell Physiol.

[34] Newton, A. C., Bootman, M. D. & Scott, J. D. Second Messengers. *Cold Spring Harb Perspect Biol* **8**, (2016). 10.1101/cshperspect.a005926 PMC4968160 27481708

[35] Zhang DY, Zhang YH, Sun HY, Lau CP, Li GR (2011). Epidermal growth factor receptor tyrosine kinase regulates the human inward rectifier potassium K(IR)2.3 channel, stably expressed in HEK 293 cells. Br J Pharmacol.

